# Iron Stores, Hepcidin, and Aortic Stiffness in Individuals with Hypertension

**DOI:** 10.1371/journal.pone.0134635

**Published:** 2015-08-05

**Authors:** Luca Valenti, Alessandro Maloberti, Stefano Signorini, Marta Milano, Francesca Cesana, Fabrizio Cappellini, Paola Dongiovanni, Marianna Porzio, Francesco Soriano, Maura Brambilla, Giancarlo Cesana, Paolo Brambilla, Cristina Giannattasio, Silvia Fargion

**Affiliations:** 1 Internal Medicine and Metabolic Diseases, Fondazione IRCCS Ca’ Granda Ospedale Policlinico, Università degli Studi di Milano, Milan, Italy; 2 Cardiology IV, De Gasperi Department, Niguarda Ca’ Granda Hospital, and Medicine Department, Università Milano Bicocca, Milan, Italy; 3 Laboratory Medicine, Desio Hospital, Università Milano Bicocca, Milan, Italy; 4 General Direction, Fondazione IRCCS Ca’ Granda Ospedale Policlinico, Research Centre on Public Health, University Milano Bicocca, Milan, Italy; Lady Davis Institute for Medical Research/McGill University, CANADA

## Abstract

**Background & Aims:**

Iron accumulation within the arterial wall has been hypothesized to promote atherosclerosis progression. Aim of this study was to evaluate whether the hormone hepcidin and iron stores are associated with arterial stiffness in subjects with essential hypertension.

**Methods:**

Circulating hepcidin, ferritin, and mutations in the hemochromatosis gene were compared between subjects included in the first vs. third tertile (n=284 each) of carotid-femoral pulse wave velocity (PWV) in an unselected cohort of patients with arterial hypertension.

**Results:**

At univariate logistic regression analysis, high PWV was associated with higher ferritin levels (p=0.010), but lower hepcidin (p=0.045), and hepcidin ferritin/ratio (p<0.001). Hemochromatosis mutations predisposing to iron overload were associated with high PWV (p=0.025). At multivariate logistic regression analysis, high aortic stiffness was associated with older age, male sex, lower BMI, higher systolic blood pressure and heart rate, hyperferritinemia (OR 2.05, 95% c.i. 1.11-3.17 per log ng/ml; p=0.022), and lower circulating hepcidin concentration (OR 0.29, 95% c.i. 0.16-0.51 per log ng/ml; p<0.001). In subgroup analyses, high PWV was associated with indices of target organ damage, including micro-albuminuria (n=125, p=0.038), lower ejection fraction (n=175, p=0.031), cardiac diastolic dysfunction (p=0.004), and lower S wave peak systolic velocity (p<0.001). Ferritin was associated with cardiac diastolic dysfunction, independently of confounders (p=0.006).

**Conclusions:**

In conclusion, hyperferritinemia is associated with high aortic stiffness and cardiac diastolic dysfunction, while low circulating hepcidin with high aortic stiffness.

## Introduction

Atherosclerosis, the leading cause of cardiovascular disease and mortality worldwide, is a chronic inflammatory disease characterized by the progressive formation of neo-intimal lesions and lumen narrowing of affected arteries. Development of atherosclerotic lesions is caused by retention of low-density lipoprotein cholesterol within arterial intima, favored by activation of immune cells with induction of oxidative stress [1]. Hypercholesterolemia, arterial hypertension, cigarette smoke, and hyperglycemia, that is the classic cardiovascular risk factors, are major driver of atherogenesis.

Arterial stiffness, a physical phenotype of the vascular wall, can be estimated by measurement of aortic pulse wave velocity (PWV) [2]. PWV reflects the advancement of atherosclerosis, and is an independent predictor of cardiovascular disease [3, 4]. Mechanisms linking arterial stiffness with cardiovascular risk are related to the effect on cardiac afterload increasing cardiac work, and the promotion of target organ damage by facilitation of the transmission of pulse waves to the microcirculation [5, 6]. Blood pressure and aging are established factors contributing to arterial stiffening, but inflammation may be involved through induction of oxidative stress [7].

However, even in patients with hypertension there is a great variability in the progression of arterial damage, and in a considerable proportion of cases vascular disease is not fully explained by classic risk factors. Iron accumulation in macrophages within the arterial wall has been hypothesized to represent a novel determinant of atherosclerosis progression by inducing oxidative stress and the release of proinflammatory mediators [8, 9]. Indeed, excess body iron stores are frequently detected in subjects with insulin resistance and related metabolic alterations [10]. Furthermore, circulating ferritin, a marker of iron accumulation and inflammation, has been linked with increased risk of carotid plaques development [11]. The hepatic hormone hepcidin regulate body iron fluxes by determining intracellular retention of this metal and reduced intestinal absorption by downregulation of the cellular exporter Ferroportin-1 [12]. Hepcidin secretion is induced by elevated body iron stores and inflammation [13]. Therefore, hepcidin may favor atherosclerosis by determining iron trapping within macrophages in atherosclerotic plaques [8]. In keeping with this hypothesis, circulating hepcidin levels correlated with the macrophage chemokine CCL2 and common carotid arteries intima media thickness (CCA-IMT) in individuals with metabolic syndrome alterations [14]. On the other hand, hepcidin may also exert anti-inflammatory activities [15].

Aim of this study was therefore to evaluate whether body iron stores and circulating hepcidin are associated with arterial stiffness in individuals with arterial hypertension.

## Materials and Methods

### Study design

The study protocol conformed to the ethical guidelines of the 1975 Declaration of Helsinki, and was approved by Institutional Ethics Review Committees of the Institution involved (Fondazione IRCCS Ca' Granda Ospedale Policlinico and Università degli Studi Milano Bicocca, all in Milan Italy). All participants provided informed written consent.

Aim of the study was to evaluate whether serum hepcidin and iron stores were associated with high vs. low PWV (third vs. first tertile) in a previously described cohort of 827 consecutive unselected patients with arterial hypertension for whom serum and DNA samples are available [16].

### Patients

From September 2006 to October 2008 827 consecutive outpatients aged 18–80 years old, followed at the Hypertension Center of San Gerardo Hospital, Monza, Italy, for essential hypertension with adequate blood pressure control were enrolled.

For the purpose of this study, subjects in the upper and lower PWV tertiles were selected, obtaining two groups of 284 subjects each. Exclusion criteria were age younger than 18 years old, pregnancy, secondary hypertension, stage 4–5 chronic kidney and advanced pulmonary disease, substance abuse, a history of cancer or of cardiovascular events in the month before the study.

For all subjects, a comprehensive medical history was collected and complete physical examination was performed. Height and weight were obtained to calculate the patient body mass index (BMI) and waist circumference was assessed halfway between the lower ribs and the iliac crest. Fasting serum glucose, total cholesterol, high-density lipoprotein (HDL), low-density lipoprotein (LDL) cholesterol, triglycerides, creatinine, interleukin-18 (IL-18), Serum Amyloid A (SAA) e High Sensitivity C-reactive Protein (CRP) were measured.

### Measurement of pulse wave velocity (PWV)

Aortic stiffness was evaluated by estimating PWV between the ipsilateral carotid and the femoral arteries with the patient in the supine position. The pressure pulse waveforms were simultaneously obtained at the two arterial sites on the right side using an automatic device (Complior, Colson; Alam Medical, Paris, France) and their distance (between the hip and neck) calculated by a rigid ruler. Measure was corrected by a 0.8 factor accordingly to the PWV measurement methods consensus document [17].

The mean of two acquisitions was used for the analysis. The intra-session within- and between- operator variability of PWV were 2% and to 4%, respectively. The corresponding value for the inter-session between- operator variability was 4% [16].

### Hepcidin, ferritin and cytokines measurement

Hepcidin-25 serum levels were measured by Liquid Chromatography-Mass Spectrometry (LC-MS) on the LTQ analyzer (Thermo Scientific). The limit of detection (LOD) and limit of quantitation (LOQ) were 0.5 ng/mL and 1.0 ng/mL, respectively. Linearity of quantification was confirmed in the following range: 1.– 500 ng/mL.

Serum Ferritin was measured by the COBAS 8000 automated analyzer (Roche Diagnostics) by ECLIA (Electro-Chemiluminescence ImmunoAssay) (LOD = 0.50 ng/mL, linearity 0.5–2000 ng/mL). Hyperferritinemia was defined as ferritin levels higher than 240 ng/ml in females and 320 ng/ml in males (reference values for the Italian population).

Serum IL-6 and IL-18 were measured by BEP 2000 the automatic analyzer (Siemens Diagnostics) by ELISA, SAA by immunonephelometry (N Latex SAA, sensitivity 0.7 mg/L) by the BN II Analyzer (Siemens Diagnostics), hs-PCR was measured by the COBAS 8000 automated analyzer (Roche Diagnostics) by immunoturbidimetry.

### Evaluation of target organ damage

Proteinuria was quantified in a subset of 126 subjects (65 in the upper and 61 in the lower PWV tertiles) on urines collected in the preceding 24 hours.

CCA-IMT was measured in a subgroup of 175 individuals (85 in the upper and 90 in the lower PWV tertiles). With the subject supine and the neck in partial extension, right CCA was scanned by the Philips Sonos 5500 ultrasonography device [18]. Two-dimensional echocardiograms were performed by an experienced cardiologist using a dedicated ultrasound machine (SONOS 5500; Philips Healthcare, MA, USA with an ultrasound transducer of 2.5MHz.). The following parameters were recorded: left ventricular end-diastolic diameter, inter-ventricular septum, posterior wall thickness and ejection fraction by the Simpson method., left ventricular mass (calculated using the Deveraux formula). Pulsed Doppler was placed on the mitral anulus and the trans-mitral flow was evaluated in order to measure diastolic function parameters (E/A ratio: early/atrial component of ventricular filling). Two-dimensional loops with the superimposed color-coded tissue doppler images map of four-chamber views with pulsed doppler signal on the later left ventricular wall was used for the analysis of the later wall motion signal and to measure absolute values of longitudinal velocity (i.e. the peak systolic velocity S').

### Genetic analysis

DNA was extracted from peripheral blood by phenol—chloroform method. Success rate in extracting DNA was 100% for each study group. *HFE* genotype (C282Y and H63D variants) was assessed by sequence allele specific PCR as previously described [19]. Random samples were confirmed by direct sequencing. Quality controls were performed to verify the reproducibility of the results. Valid genotypic data were obtained for the totality of subjects analyzed. Presence of *HFE* genotypes at risk of iron overload was defined according to previous literature data [20].

### Statistical analysis

For descriptive statistics, continuous traits were summarized as means±SD. Highly skewed variables—such as circulating hepcidin-25 and ferritin concentration—were summarized as medians and interquartile range. Categorical variables are shown as percentages. Analyses were performed by fitting data to generalized linear regression models. Logistic regression models were fit to examine binary traits (high vs. low PWV). Hepcidin and ferritin levels were log-transformed before entry into the models.

Statistical analyses were carried out with JMP 11.0 (SAS Institute, Cary, NC). A two-sided P value <0.05 was considered statistically significant.

## Results

### Clinical features of individuals with hypertension according to arterial stiffness

The clinical features of patients stratified according to PWV tertiles (third vs. first tertile) are shown in Table 1. As expected, subjects with high stiffness had thicker CCA-IMT, older age, were more frequently males and active smokers, had larger abdominal circumference, higher SBP and heart rate, and among inflammatory markers, higher interleukin-18 levels (p<0.05). At multivariate logistic regression analysis corrected for demographic features (age and sex), only age, sex, SBP, and heart rate remained associated with high PWV.

**Table 1:** Clinical features of 568 Italian patients with essential hypertension stratified according to common carotid arteries stiffness (third vs. first tertile).

	Stiffness					
	High (n = 284)	Low (n = 284)	OR (95% CI)	p	OR (95% CI)*	p*
PWV, m/sec	10±2.5	7.4±1.6	2.40 (2.08–2.80)	<0.001	2.12 (1.84–2.49)	<0.001
CCA-IMT, mm ^	0.8 ± 0.2	0.7 ± 0.2	19.4 (3.1–146)	0.001	12.6 (0.60–47)	0.14
Age, per 10 years	63±11	53±11	2.38 (2.00–2.88)	<0.001	2.44 (2.04–2.96)	<0.001
Sex, male	182 (64)	139 (49)	1.88 (1.34–2.63)	<0.001	1.96 (1.35–2.87)	<0.001
Currently smoking, yes	32 (11)	47 (17)	0.57 (0.36–0.91)	0.019	0.67 (0.37–1.12)	0.13
Waistline, cm	96±12	94±13	1.02 (1.00–1.03)	0.025	1.00 (0.99–1.01)	0.82
BMI, Kg/m^2^	27±4	27±5	0.97 (0.83–1.01)	0.21	0.96 (0.91–1.01)	0.10
Hb, g/dl	13.9±2.2	13.7±2.7	1.06 (0.82–1.36)	0.42	0.98 (0.77–1.22)	0.86
Glucose, mg/dl	101±22	94±28	1.00 (0.99–1.01)	0.12	1.00 (0.99–1.01)	0.31
Triglycerides, mg/dl	112±59	95±47	1.01 (1.00–1.01)	0.064	1.01 (1.00–1.01)	0.11
Total cholesterol, mg/dl	194 ± 36	197 ± 33	1.00 (0.99–1.01)	0.60	1.00 (1.00–1.00)	0.65
HDL, mg/dl	55±18	58±14	0.99 (0.97–1.01)	0.36	1.00 (0.97–1.02)	0.93
SBP, per 10 mmHg	140±21	126±19	1.04 (1.03–1.05)	<0.001	1.03 (1.02–1.05)	<0.001
DBP, mmHg	80±14	79±13	1.01 (0.99–1.02)	0.29	1.02 (1.00–1.03)	0.007
Heart rate, bpm	72±12	71±13	1.01 (1.00–1.02)	0.10	1.03 (1.01–1.05)	<0.001
IL18, pg/ml	243 {187–308}	219 {175–280}	3.91° (1.56–10.1)	0.004	1.03° (0.35–3.03)	0.97
SAA, mg/l	5.3 {3.2–8.4}	5.4 {3.6–9.2}	0.88° (0.57–1.35)	0.56	0.90° (0.56–1.47)	0.68
hs-CRP, mg/l	1.4 {0.7–3.3}	1.6 {0.8–3.9}	0.82 (0.58–1.16)	0.26	0.90 (0.60–1.37)	0.59

Data are shown as means±SD, prevalence (% value), median {interquartile range}, as required. CCA: common carotid arteries; PWV: pulse wave veIocity; IMT: intima-media thickness, HDL: high-density lipoprotein cholesterol; SBP: systolic blood pressure; DBP: diastolic blood pressure; bpm: beats per minute; IL18: interleukin-18, SAA: serum amyloid A protein, hs-CRP: high sensitivity C reactive protein. Comparisons were made by fitting data to logistic regression models. OR: odds ratio for high vs. low CCA stiffness; CI: confidence intervals.

*Adjusted for age and sex.

^Available in 175 individuals.

° per 1 log increase.

### Iron parameters and hepcidin determinants

Iron parameters according to PWV tertiles are shown in Table 2. Ferritin and hepcidin levels were generally within the normal range, and the frequency distribution of *HFE* genotypes (reported in S1 Table) were in line with the expected prevalence in Northern Italy and did not violate Hardy-Weinberg equilibrium.

**Table 2:** Iron status in 568 Italian patients with essential hypertension stratified according to common carotid arteries stiffness (third vs. first tertile).

	Stiffness					
	High (n = 284)	Low (n = 284)	OR (95% CI)	p	OR (95% CI)*	p*
Ferritin, ng/ml	152 {82–269}	137 {60–141}	1.63° (1.13–2.83)	0.010	0.94° (0.57–1.55)	0.82
Hyperferritinemia, yes	53 (20)	36 (13)	1.53 (1.00–2.50)	0.05	1.20 (0.70–2.06)	0.52
Hepcidin, ng/ml	27 {13–49}	32 {15–59}	0.64° (0.41–0.99)	0.045	0.39° (0.23–0.65)	<0.001
Hepcidin/ferritin, ratio	0.18 {0.09–0.35}	0.27 {0.13–0.48}	0.73° (0.61–0.87)	<0.001	0.77° (0.63–0.94)	0.010
*HFE* genotype at risk^	13 (5)	4 (1)	3.30 (1.15–11.9)	0.025	3.53 (1.05–14.5)	0.041

Data are shown as prevalence (% value), median {interquartile range}, as required. Comparisons were made by fitting data to logistic regression models. OR: odds ratio for high vs. low CCA stiffness; CI: confidence intervals.

* Adjusted for age and sex.

° per 1 log increase;

^ Defined as C282Y/C282Y, C282Y/H63D, or H63D/H63D vs. H63D/wild-type and wild-type/wild-type [20].

**Fig 1:**
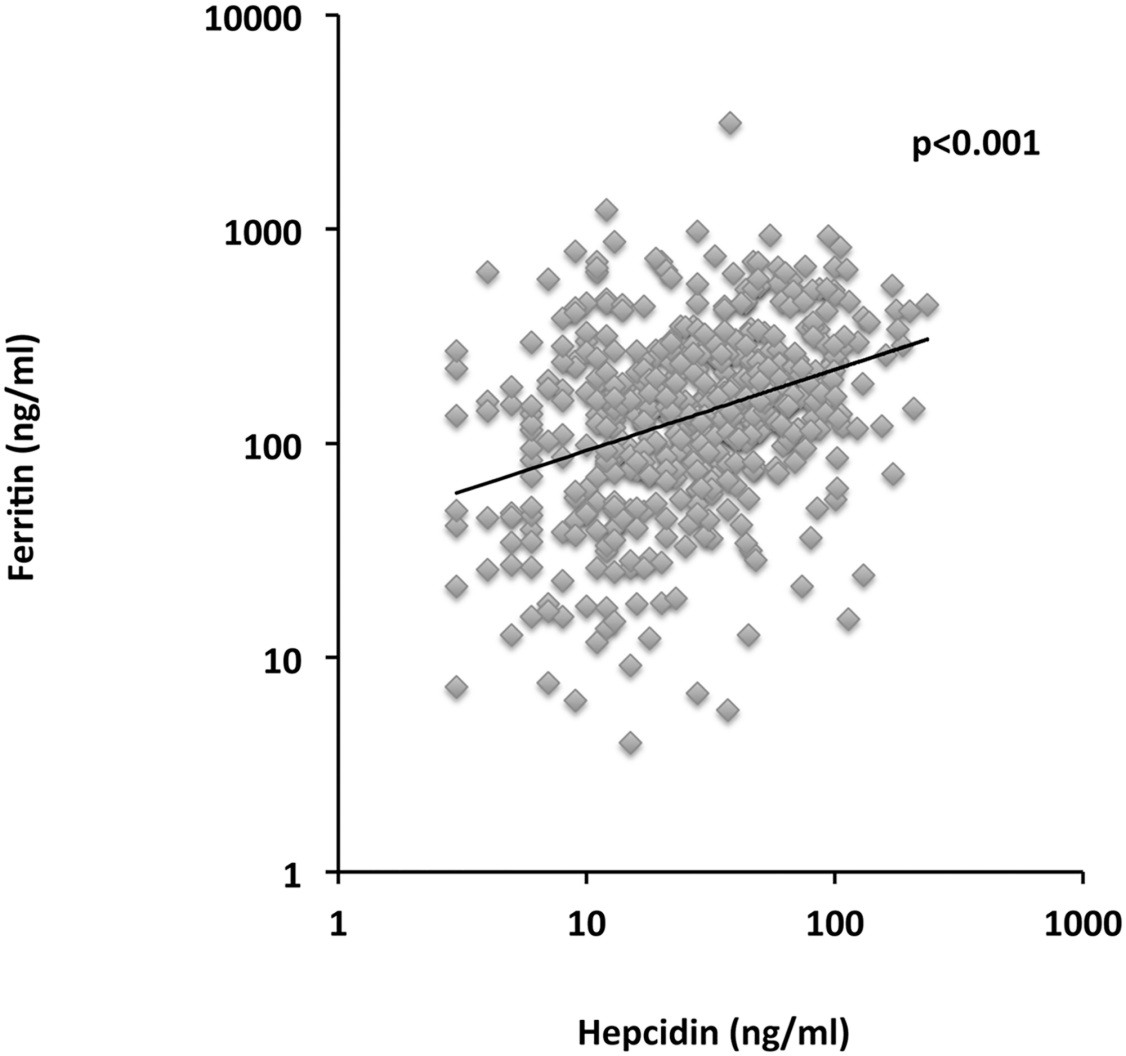
Correlation between ferritin and hepcidin levels (p<0.001).

As expected, the hepcidin/ferritin ratio was lower in patients carrying *HFE* genotypes at risk of iron overload (0.10, IQR 0.07–0.21 vs. 0.22, IQR 0.11–0.41 ng/ml; p = 0.001). Independent predictors of hepcidin levels at multivariate generalized linear model are shown in Table 3 and in Fig 1. Ferritin was the main determinant of hepcidin levels also in the present cohort of patients with hypertension (estimate per 1 log increase 1.60±0.07; p<0.001), but no other independent predictors could be identified. In particular, ferritin was not significantly associated with any inflammatory marker.

**Table 3:** Predictors of circulating hepcidin in 568 patients with arterial hypertension. We included variables available for all patients evaluated, when p≤0.10 for association with ferritin levels at univariate analysis.

	Estimate	SE	p value	Estimate*	SE*	p value*
Age, per 10 years	+0.03	0.01	0.025	-0.02	0.02	0.28
Sex, male	+0.04	0.01	0.019	+0.01	0.01	0.31
IL18, log pgml	+0.15	0.09	0.10	-0.04	0.09	0.68
Ferritin, log ng/ml	+0.31	0.04	<0.001	-	-	-

Comparisons were made by fitting data to generalized linear models. OR: odds ratio; c.i.: confidence interval;

* Adjusted for serum ferritin.

### Association of iron parameters with arterial stiffness

At univariate logistic regression analysis (Table 2, left panel), high stiffness was associated with higher ferritin levels (p = 0.010), but lower hepcidin (p = 0.045), and lower hepcidin/ferritin ratio (p<0.001). The presence of *HFE* genotypes predisposing to iron overload was associated with high PWV (p = 0.025).

At multivariate logistic regression analysis corrected for age and sex (Table 2, right panel), lower hepcidin (p<0.001), lower hepcidin/ferritin ratio (p = 0.01), and *HFE* genotypes at risk of iron overload (p = 0.041) remained associated with high PWV.

### Independent predictors of arterial stiffness

Independent predictors of high PWV at multivariate logistic regression analysis are shown in Table 4. In the full model adjusted for the independent determinants identified above, high aortic stiffness was associated with older age, male sex, lower BMI, higher SBP and heart rate, hyperferritinemia (OR 2.05, 95% c.i. 1.11–3.17 per log ng/ml; p = 0.022), and lower circulating hepcidin (OR 0.29, 95% c.i. 0.16–0.51 per log ng/ml; p<0.001). The association between hepcidin and high arterial stiffness was significant both in males (adjusted OR 0.35, 95% c.i. 0.17–0.68 per log ng/ml; p = 0.002), and in females (adjusted OR 0.33, 95% c.i. 0.12–0.88 per log ng/ml; p = 0.026). Independent determinants of PWV in patients stratified by the presence of type 2 diabetes mellitus (T2DM) are shown in S2 Table. In patients without T2DM (n = 498), high PWV remained borderline associated with hyperferritinemia (adjusted OR 1.96, 95% c.i. 1.00–3.94; p = 0.05) and associated with hepcidin (adjusted OR 0.26, 95% c.i. 0.13–0.48 per log ng/ml; p<0.001).

**Table 4:** Independent predictors of elevated arterial stiffness at multivariate logistic regression analysis in 568 Italian patients with essential hypertension.

	OR	95% c.i.	p value
Age, per 10 years	2.67	2.14–3.40	<0.001
Sex, male	2.09	1.29–3.40	0.002
BMI, Kg/m^2^	0.91	0.86–0.97	0.003
SBP, per 10 mmHg	1.47	1.28–1.70	<0.001
Heart rate, per 10 bpm	1.30	1.07–1.59	0.007
Hepcidin, log ng/ml	0.29	0.16–0.51	<0.001
Hyperferritinemia, yes	2.05	1.11–3.87	0.022

Comparisons were made by fitting data to a logistic regression model, adjusted for age, sex, BMI, SBP, heart rate, hepcidin levels, and presence of hyperferritinemia. OR: odds ratio; c.i.: confidence interval; BMI: body mass index; SBP: systolic blood pressure; bpm: beats per minute.

### Hepcidin and target organ damage

In the subset of patients with available data, micro-albuminuria was higher in individuals in the higher vs. those in the lower PWV tertile (median 5.5, IQR 1.6–12.2 vs. 3.6, IQR 0.2–9.4 mg/day; estimate 0.32±0.13; p = 0.038). At generalized linear regression, there was no significant association between hepcidin or ferritin levels with albuminuria (p>0.1).

Cardiac function parameters stratified by PWV tertiles are shown in Table 5. Concerning cardiac function parameters, the ejection fraction (p = 0.025), E/A ratio (p = 0.002), and S wave peak systolic velocity (p<0.001) were lower in individuals in the higher vs. those in the lower PWV tertile. High PWV was not significantly associated with left ventricular end diastolic diameter, deceleration time, thickness of the inter-ventricular septum, and left ventricular mass index (p>0.1 for all), while there was a trend for association with posterior wall thickness (p = 0.085). At multivariate logistic regression analysis adjusted for sex and age, only S wave peak systolic velocity remained associated with high PWV (p<0.001).

**Table 5:** Heart function parameters in 175 Italian patients with essential hypertension stratified according to common carotid arteries stiffness (third vs. first tertile).

	Stiffness					
	High (n = 85)	Low (n = 90)	OR (95% CI)	p	OR (95% CI)*	p*
LVM index, g	110±26	105±26	1.01 (0.99–1.02)	0.23	1.01 (0.99–1.02)	0.45
IVS, cm	1.07±0.15	1.03±0.14	5.00 (0.61–43.8)	0.13	3.5 (0.30–11.1)	0.36
PWT, cm	1.00±0.13	0.96±0.12	5.50 (0.79–43.6)	0.085	2.37 (0.14–45.0)	0.54
LVEDD, cm	4.72±0.54	4.61±0.49	1.53 (0.85–2.84)	0.15	1.19 (0.57–2.45)	0.64
Ejection fraction, %	61.4±6.4	63.1±3.8	0.93 (0.86–0.99)	0.025	0.94 (0.88–1.01)	0.11
E/A, ratio	0.99±0.39	1.15±0.31	0.23 (0.08–0.61)	0.002	0.64 (0.19–1.77)	0.40
S wave peak systolic velocity, cm/s	9.6±2.7	12.5±3.4	0.73 (0.65–0.82)	<0.001	0.77 (0.67–0.88)	<0.001

Data are shown as prevalence (% value), median {interquartile range}, as required. Comparisons were made by fitting data to logistic regression models. OR: odds ratio for high vs. low CCA stiffness; CI: confidence intervals.

* Adjusted for age and sex. LVMI: left ventricular mass; PWT: posterior wall thickness; IVS: inter-ventricular septum.

Hepcidin levels were inversely correlated with the E/A ratio (estimate per 1 log increase -0.16±0.07; p = 0.031; S1A Fig), but not with the EF (estimate 0.44±0.14; p = 0.70) nor S wave peak systolic velocity (estimate -1.15±0.73; p = 0.13). Ferritin levels were inversely correlated with E/A ratio (estimate per 1 log increase -0.20±0.06; p = 0.001; S1B Fig) and S wave peak systolic velocity (estimate -1.38±0.57; p = 0.018), but not with ejection fraction (estimate -0.04±0.92; p = 0.97). At multivariate generalized linear model analysis (shown in S3 Table), cardiac diastolic function (estimated by the E/A ratio) was negatively associated with circulating ferritin (p = 0.006), but not with hepcidin levels (p = 0.98). No independent association was observed between ferritin and systolic function (peak S wave velocity; not shown).

## Discussion

To test whether elevated iron stores and circulating hepcidin favor the progression of atherosclerosis [8, 9, 11, 14], in this study we examined whether ferritin and hepcidin were associated with aortic stiffness in a cross-sectional cohort of Italian patients with hypertension. We found that hyperferritinemia was associated with increased, while hepcidin with decreased PWV.

The association of hyperferritinemia with increased arterial stiffness is consistent with the hypothesis that elevated body iron stores are associated with atherosclerosis progression [9–11, 14]. Indeed, in the present cohort hyperferritinemia did not reflect subclinical inflammation. On the other hand, hyperferritinemia is frequently observed in individuals with arterial hypertension [10, 21], and in subjects with metabolic alterations ferritin was previously associated with increased CCA-IMT, presence of carotid plaques, and circulating CCL2 levels, a macrophage chemokine involved in the progression of atherosclerosis [11, 14]. In addition, in patients with hereditary hemochromatosis iron depletion improved radial artery thickness and distensibility [22]. In this study, we also observed an inverse correlation between circulating ferritin and left ventricular diastolic function (estimated by the E/A ratio), independent of other risk factors. These results were obtained in a limited subgroup of patients with complete characterization and should be further confirmed. However, data are consistent with the hypothesis that increased body iron stores contribute to the pathogenesis of early diastolic dysfunction. Indeed, cardiac disease and restrictive cardiomyopathy represent a typical manifestation of body iron overload favored by oxidative stress [23].

Conversely, we observed an inverse association between circulating hepcidin and PWV. Therefore, results do not support a role of hepcidin in determining the progression of atherosclerosis in large conductance vessels. This was previously hypothesized based on data suggesting that hepcidin mediates iron accumulation within the arterial plaque [24], and in particular in macrophages which phagocyte red blood cells deriving by intra-plaque hemorrhages [8, 14, 25, 26]. Hepcidin was also shown to correlate with arterial plaques in postmenopausal women [27], to inhibit *in vitro* cholesterol efflux from macrophages and foam cells formation [28], and high hepcidin predicted cardiovascular events in hemodialysis patients [29]. Furthermore, in hemodialysis patients hepcidin has been also directly associated with increased carotid PWV [30], and we previously reported a positive association between hepcidin levels and carotid damage in high-risk patients [11].

On the contrary, in the present study higher hepcidin was inversely associated with aortic stiffening, independently of the degree of iron accumulation. Differently from previous studies, which estimated PWV of brachial arteries or focused on CCA damage, thereby evaluating mainly resistive vessels, we specifically measured stiffness of the aorta, the major conductance elastic artery. In keeping with the association of hepcidin with lower aortic stiffness, hepcidin has also been reported to exert anti-inflammatory activities by inhibiting cytokine signaling [15], possibly resulting in protection from aortic fibrosis [31]. It could be speculated that, due to the different anatomical structure and cell composition, hepcidin has different biological effects in the aorta as compared to resistive peripheral arteries, contributing to explain the observed discrepancies.

Alternatively, since insulin signaling has been reported to decrease hepcidin release [32], lower serum hepcidin in individuals with high PWV may reflect more severe insulin resistance in this high-risk group. Although type 2 diabetes was not associated with hepcidin levels, we cannot rule out the aforementioned hypothesis because it was not possible to evaluate fasting insulin levels in all patients.

Despite the relatively high number of subjects evaluated, the study is limited by the cross-sectional design, so that we cannot exclude that hepcidin favors the formation of less stiff, but unstable plaques resulting in increased cardiovascular risk. In addition, we could not evaluate transferrin saturation, which is another determinant of hepcidin release, and the study was not powered enough to correct the association of iron parameters with PWV for the use of specific anti-hypertensive drugs. On the other hand, supporting the reliability of our findings, we observed a strong association between hepcidin levels and serum ferritin. In addition, we validated the association of arterial stiffness with other known determinants, including age, male sex, systolic blood pressure, and heart rate. Even if apparently counterintuitive, a negative association between high BMI on aortic PWV, after controlling for peripheral SBP, has previously been reported [33, 34]. This “protective” effect of overweight on aortic stiffness was related to modification of aortic walls, different mechanisms of adaptation to increased blood pressure, or volume overload. Furthermore, obesity has also been previously associated with increased brachial artery compliance in young normotensive subjects [35]. This finding indicates that not all risk factors for vascular events, possibly including high hepcidin levels, are invariably associated with increased aortic stiffness.

In conclusion, hyperferritinemia is associated with high aortic stiffness and cardiac diastolic dysfunction (E/A ratio), and low circulating hepcidin is associated with high aortic stiffness.

## Supporting Information

S1 Fig Correlation between iron parameters (hepcidin and ferritin levels) and cardiac diastolic function (E/A ratio). (DOCX)

S1 File Study database. (XLSX)

S1 Table *HFE* genotypes in 568 Italian patients stratified by common carotid arteries stiffness (third vs. third tertile). Prevalence (% values) are shown. Wt: wild-type.
(DOCX)

S2 Table Independent predictors of elevated arterial stiffness at multivariate logistic regression analysis in 568 Italian patients with essential hypertension, stratified by the presence of type 2 diabetes mellitus (T2DM). Comparisons were made by fitting data to a logistic regression model, adjusted for age, sex, BMI, SBP, heart rate, hepcidin levels, and presence of hyperferritinemia. OR: odds ratio; c.i.: confidence interval; BMI: body mass index; SBP: systolic blood pressure; bpm: beats per minute.
(DOCX)

S3 Table Independent predictors of E/A ratio in 175 Italian patients with hypertension who underwent cardiac ecocolordoppler evaluation. Comparisons were made by fitting data to a generalized linear model, adjusted for age, PWV, hepcidin, and ferritin levels. SE: standard error; PWV: pulse wave velocity.
(DOCX)
