# Supplementary material for: Iron Stores, Hepcidin, and Aortic Stiffness in Individuals with Hypertension
Source: PLoS One. 2015 Aug 5;10(8):e0134635. doi: 10.1371/journal.pone.0134635 (PMC4526526; doi:10.1371/journal.pone.0134635)
Supplement: S1 Fig — (DOCX) [file pone.0134635.s001.docx]

**Figure S1**. Correlation between iron parameters (hepcidin and ferritin levels) and cardiac diastolic function (E/A ratio).
