# Supplementary material for: Iron Stores, Hepcidin, and Aortic Stiffness in Individuals with Hypertension
Source: PLoS One. 2015 Aug 5;10(8):e0134635. doi: 10.1371/journal.pone.0134635 (PMC4526526; doi:10.1371/journal.pone.0134635)
Supplement: S1 Table — Prevalence (% values) are shown. Wt: wild-type. (DOCX) [file pone.0134635.s003.docx]

**Table S1**. *HFE* genotypes in 568 Italian patients stratified by common carotid arteries stiffness (third vs. third tertile).

|  | High stiffness  n=284 | Low stiffness  n=284 |
| --- | --- | --- |
| *HFE* GENOTYPE |  |  |
| wt/wt | 192 (0.676) | 186 (0.654) |
| H63D/wt | 71 (0.250) | 82 (0.288) |
| C282Y/wt | 8 (0.028) | 12 (0.042) |
| H63D/H63D | 9 (0.031) | 3 (0.010) |
| C282Y/H63D | 2 (0.007) | 0 |
| C282Y/C282Y | 2 (0.007) | 1 (0.003) |

Prevalence (% values) are shown. Wt: wild-type.
