# Supplementary material for: Iron Stores, Hepcidin, and Aortic Stiffness in Individuals with Hypertension
Source: PLoS One. 2015 Aug 5;10(8):e0134635. doi: 10.1371/journal.pone.0134635 (PMC4526526; doi:10.1371/journal.pone.0134635)
Supplement: S2 Table — Comparisons were made by fitting data to a logistic regression model, adjusted for age, sex, BMI, SBP, heart rate, hepcidin levels, and presence of hyperferritinemia. OR: odds ratio; c.i.: confidence interval; BMI: body mass index; SBP: systolic blood pressure; bpm: beats per minute. (DOCX) [file pone.0134635.s004.docx]

**Table S2.** Independent predictors of elevated arterial stiffness at multivariate logistic regression analysis in 568 Italian patients with essential hypertension, stratified by the presence of type 2 diabetes mellitus (T2DM).

|  | Without T2DM  n = 498 | | | With T2DM  n = 70 | | |
| --- | --- | --- | --- | --- | --- | --- |
|  | OR | 95% c.i. | p value | OR | 95% c.i. | p value |
| Age, per 10 years | 2.73 | 2.15-3.56 | <0.001 | 2.15 | 0.97-5.67 | 0.09 |
| Sex, male | 1.99 | 1.18-3.38 | 0.001 | 4.07 | 0.87-22.5 | 0.08 |
| BMI, Kg/m^2^ | 0.89 | 0.83-0.96 | 0.001 | 1.02 | 0.84-1.24 | 0.83 |
| SBP, per 10 mmHg | 1.58 | 1.36-1.86 | <0.001 | 1.11 | 0.84-1.61 | 0.49 |
| Heart rate, per 10 bpm | 1.26 | 1.03-1.56 | 0.03 | 1.75 | 0.96-3.40 | 0.08 |
| Hepcidin, log ng/ml | 0.26 | 0.13-0.48 | <0.001 | 0.32 | 0.05-1.93 | 0.22 |
| Hyperferritinemia, yes | 1.96 | 1.00-3.94 | 0.05 | 2.38 | 0.46-14.51 | 0.31 |

Comparisons were made by fitting data to a logistic regression model, adjusted for age, sex, BMI, SBP, heart rate, hepcidin levels, and presence of hyperferritinemia. OR: odds ratio; c.i.: confidence interval; BMI: body mass index; SBP: systolic blood pressure; bpm: beats per minute.
