# Supplementary material for: Iron Stores, Hepcidin, and Aortic Stiffness in Individuals with Hypertension
Source: PLoS One. 2015 Aug 5;10(8):e0134635. doi: 10.1371/journal.pone.0134635 (PMC4526526; doi:10.1371/journal.pone.0134635)
Supplement: S3 Table — Comparisons were made by fitting data to a generalized linear model, adjusted for age, PWV, hepcidin, and ferritin levels. SE: standard error; PWV: pulse wave velocity. (DOCX) [file pone.0134635.s005.docx]

**Table S3**. Independent predictors of E/A ratio in 175 Italian patients with hypertension who underwent cardiac ecocolordoppler evaluation.

|  | Estimate | SE | p value |
| --- | --- | --- | --- |
| Age, per 10 years | -0.10 | 0.02 | <0.001 |
| PWV, high | -0.02 | 0.03 | 0.35 |
| Hepcidin, log ng/ml | -0.00 | 0.08 | 0.98 |
| Ferritin, log ng/ml | -0.22 | 0.08 | 0.006 |

Comparisons were made by fitting data to a generalized linear model, adjusted for age, PWV, hepcidin, and ferritin levels. SE: standard error; PWV: pulse wave velocity.
